# Mycobiomes of the Ocular Surface in Bacterial Keratitis Patients

**DOI:** 10.3389/fopht.2022.894739

**Published:** 2022-05-05

**Authors:** Rajagopalaboopathi Jayasudha, Sama Kalyana Chakravarthy, Gumpili Sai Prashanthi, Savitri Sharma, Prashant Garg, Somasheila I. Murthy, Sisinthy Shivaji

**Affiliations:** ^1^ Prof. Brien Holden Eye Research Centre, L V Prasad Eye Institute, Hyderabad, India; ^2^ The Cornea Institute, L V Prasad Eye Institute, Hyderabad, India

**Keywords:** bacterial keratitis, ocular mycobiome, conjunctiva, cornea, dysbiosis, NGS

## Abstract

Inflammation of the cornea is known as keratitis, and bacteria, fungi, protozoans, and viruses are the etiological agents of this disease. Delayed treatment of keratitis could result in loss of vision and, under certain severity conditions, the removal of an eye and its associated structures. In the current study, the ocular surface (conjunctiva and cornea) mycobiomes of individuals with bacterial keratitis were compared with the ocular mycobiome (conjunctiva) of healthy individuals, free of any ocular morbidity. Mycobiomes were generated through NGS approach using conjunctival swabs and corneal scrapings as the source of DNA from which ITS2 was amplified and sequenced, as a proxy to identify fungi. The results indicated significant changes in the alpha-diversity indices and in the abundance at the phylum and genera level. Hierarchical clustering using a heatmap showed that the mycobiomes were different. Furthermore, NMDS plots also differentiated the mycobiomes in the three cohorts, implying dysbiosis in the mycobiomes of the conjunctivae and corneal scrapings of bacterial keratitis individuals compared to control individuals. A preponderance of negative interactions in the hub genera in the conjunctival swabs of bacterial keratitis individuals compared to healthy controls further re-emphasized the differences in the mycobiomes. The dysbiotic changes at the genera level in conjunctivae and corneal scrapings of bacterial keratitis individuals are discussed with respect to their possible role in causing or exacerbating ocular surface inflammation. These results demonstrate dysbiosis in the ocular mycobiome in bacterial keratitis patients compared to healthy controls for the first time.

## Introduction

The ocular surface harbors a variety of microorganisms (1), which together constitute the microbiome. The conventional cultivable approach has revealed the presence of several bacteria (such as coagulase negative staphylococcus, *Staphylococcus aureus*, *Propionibacterium* sp., *Micrococcus* sp., and *Corynebacterium* sp.) (2–8) and fungi (such as *Curvularia* sp., *Fusarium* sp., *Alternaria* sp., *Saccharomyces cerevisiae*, *Helminthosporium* sp., *Aspergillus flavus, A*. *niger*, *Penicillium* sp., *Candida guilliermondii*, *C. parapsilosis*, *C. albicans*, *Rhodotorula rubra*, and *Hormodendrum* sp.) (9–13) on the ocular surface. Furthermore, next-generation sequencing (NGS) technology, involving amplicon sequencing of the internal transcribed spacer 2 (ITS2), established that the ocular mycobiomes are diverse and abundant (14, 15). In the NGS method, fungi were identified in majority of the swabs (73.5%) and 65 genera were identified including *Aspergillus*, *Setosphaeria, Haematonectria*, and *Malassezia* in all the sampled eyes (15). It was also confirmed by Prashanthi et al. (14) that a similar proportion of fungi (74%) were also detected in the ocular surface mycobiomes from individuals with fungal keratitis as in healthy individuals. In ocular diseases, such as keratitis, conjunctivitis, blepharitis, and endophthalmitis (16), fungi are the disease-causing agents. Thus, in the diseased state, the ocular surface fungal mycobiome is likely to be altered compared to that existing in the healthy control eyes (17). In our earlier studies, dysbiosis in gut microbiomes and mycobiomes was observed in patients with ocular diseases like uveitis (18, 19), in both bacterial and fungal keratitis (20) and also in diabetic retinopathy (21, 22). Similar studies on dysbiotic changes in the ocular surface microbiomes are only few and limited to blepharitis patients (23), contact lens wearers (24), Stevens**–**Johnson syndrome patients (25), fungal keratitis patients (14, 26), and bacterial keratitis (BK) patients (27). These findings implicated the involvement of ocular surface microbiome/mycobiome in the pathogenesis of ocular diseases.

Globally, 6–8 million people are affected by bilateral blindness due to corneal disease, the majority due to sequelae from infectious keratitis (28). Trauma is the cause of keratitis in 47.6% of affected individuals (29). Patients with keratitis share several common symptoms that affect the eye such as redness, pain, sensitivity to light, blurred vision, foreign body feeling tearing, and discharge. These symptoms are common irrespective of whether bacteria, fungi, protozoa, or viruses are the causative agents of keratitis. Microbiological studies following the culture of corneal scrapings are the gold standard for determining the etiology of infectious keratitis caused by bacteria, fungi, protozoa, etc. However, a major problem related to identification of the causative agent of keratitis is that growth occurs in only 40%–60% of the cases (30–33). Thus, corneal ulcers are often treated empirically by cornea specialists, who can differentiate clinically bacterial and fungal keratitis but in fewer than 70% of cases (34), thus implying that bacteria or fungi may not be exclusively present in bacterial or fungal keratitis, respectively. In fact, keratitis, could be polymicrobial, caused by two or more organisms (e.g., bacteria–bacteria, fungus–fungus, bacteria–fungus, and fungus–protozoan) in approximately 2%–15% of all keratitis cases (35–40). Thus, in view of the relatively common occurrence of fungi as a common etiological agent of keratitis, it is important to investigate the mycobiomes of the ocular surface (conjunctiva and cornea) in the control cohort of healthy individuals and compare the mycobiomes with individuals with BK to assess whether ocular mycobiome dysbiosis is prevalent in BK patients. This study would also shed light on whether the fungal community of conjunctiva is different from that of the cornea.

## Materials and Methods

### Recruitment of Subjects

All individuals were recruited by the in-house doctors at the L V Prasad Eye Institute (LVPEI), Hyderabad, India. The recruits included healthy controls (HC) (18 men and 8 women: age range, 23–77 years; mean age, 44.15 years) without any ocular complications or disease such as dry eye disease, keratitis, uveitis, glaucoma, and retinal diseases. These 26 were recruited for investigating the conjunctival surface mycobiomes. The yearly incidence of BK in India is 2.79 per 10,000 (41). The sample size derived was 21 based on the population proportion method (90% confidence interval and 6% error). Therefore, 22 patients with microbiologically proven BK (18 men and 4 women; age range, 27–71 years; mean age, 51.54) based on microscopic examination of corneal scrapings were recruited for the study. Patients with Gram-positive and Gram-negative bacterial infections in the cornea were included in the study, but patients with fungal keratitis or mixed infections were excluded. Both swabs of the conjunctivae and scrapings of the cornea were collected from the BK patients. Both HC and BK individuals did not wear contact lenses. HC individuals abstained for 3 months before sample collection from oral or topical antibacterial and antifungal medicines, corticosteroids, or non-steroidal anti-inflammatory agents. Among the 22 BK patients, 5 patients had taken topical antibiotics and 1 patient (BK-033) had taken both oral and topical antibiotics prior to recruitment for the study (see **Supplementary Table S1**). All the study participants were informed in advance about the study in a language they were familiar with, and written consent was taken prior to the collection of samples. Furthermore, the study was conducted in accordance with the Research Review Board and Ethics Committee of LVPEI, Hyderabad (Ethics Ref. No. LEC 06-14-060).

### Sample Collection

Conjunctival swabs (42) from the right and left eyes of 26 healthy individuals (HC-SW) and 22 BK patients (BK-SW, ***n*** = 22) were collected using a sterile Isohelix swab (Harrietsham, Kent, United Kingdom), which was moistened with sterile phosphate-buffered saline (PBS) (pH 7.4) just before use. Corneal scrapings from the infected eye of 22 BK patients (BK.CR, *n* = 22) were also collected using a sterile surgical blade No. 15. Proparacaine hydrochloride (0.5%) eye drops were topically applied prior to the collection of conjunctival swabs and corneal scrapings. All these procedures were described recently (27). The conjunctival swabs and corneal scrapings were stored at −80°C in PBS.

### Cultivation of Fungi

The conjunctival swabs of HC (HC-SW) and corneal scrapings of BK patients (BK-CR) were cultured on six different media (Sabouraud dextrose agar, 5% sheep blood agar, potato dextrose agar, chocolate agar, thioglycollate broth, and brain heart infusion broth) to identify the culturable microorganisms (43). The Institutional Ethics Committee approved collection of conjunctival and corneal scrapings from the BK patients, but in the HC, permission was granted only for the collection of the conjunctival swabs for ocular microbiome analysis.

### Amplicon Library Preparation and Sequencing

Conjunctival swabs and corneal scrapings were used as the source of DNA, which was used as a template to amplify the internal transcribed spacer region (ITS2), using the conserved primers ITS3 and ITS4, respectively (14). Nuclease-free sterile water was used for the preparation of all PCR reagents. The PCR controls included extracts of unused Isohelix swabs and all reagents used for DNA extraction, and none of these yielded any DNA; sequencing was also negative for fungal reads (14, 15, 27). Illumina protocol (44) was used to generate fungal amplicon libraries. Briefly, the quality of PCR products was checked on a 1.5% agarose gel; the amount of DNA quantified and subsequently the amplicon libraries were prepared using Nextera XT Index Kit (Illumina Inc.) as per the Sequencing Library preparation protocol (Part # 15044223 Rev. B). The amplicons with the adaptors were then amplified using i5 and i7 primers that add multiplexing index sequences as well as common adapters required for cluster generation (P5 and P7). The amplicon libraries were then purified by 1X AMpureXP beads, checked on Agilent DNA 1000 chip on Bioanalyzer 2100 and quantified by Qubit Fluorometer 2.0 using a Qubit dsDNA HS Assay kit (Life Technologies). For subsequent cluster generation and sequencing, the amplicon library was loaded onto Illumina platform (10–20 pM) and sequenced using 2 × 250 base pair chemistry on an Illumina HiSeq sequencer at Xcelris Genomics Pvt. Ltd., Ahmedabad, India. Paired-end sequencing allows the template fragments to be sequenced in both the forward and reverse directions on Illumina platform. The kit reagents were used in binding of samples to complementary adapter oligos on paired-end flow cell. The adapters were designed to allow selective cleavage of the forward strands after re-synthesis of the reverse strand during sequencing. The copied reverse strand was then used to sequence from the opposite end of the fragment.

### Taxonomy Assignment of Sequenced Reads

Paired-end reads of each sample were assembled using FLASH software (45). Sequences with an average Phred score of less than 25 (low quality sequences) and chimeric sequences were eliminated using Prinseq-lite (46) and Usearch61 (47), respectively. Only high-quality (HQ) reads were used for operational taxonomic unit (OTU) pickup using UNITE OTUs (ITS) version 8.3 (https://doi.org/10.15156/BIO/1264708) as available in the QIIME pipeline (48). Denovo-OTUs were classified taxonomically through Wang Classifier (49) with 80% bootstrap threshold. Sparse OTUs (<0.001% of the total number of reads) were not analyzed. Genomic DNA extraction and sequencing were performed in different batches. Batch I included 25 HC-SW, 4 BK-SW, and 2 BK-CR samples; batch II included 10 HC-SW, 3 BK-SW, and 2 BK-CR samples; batch III included 11 HC-SW and 4 BK-SW samples; and batch IV included 5 BK-SW samples. HQ reads from the above batches were analyzed together up to OTU picking and taxonomy assignment. Batch effect correction was then performed individually for each cohort using ComBat function in the SVA package (50).

### Diversity Analyses of the Mycobiomes

R-Vegan 2.4-2 package (http://vegan.r-forge.r-project.org/) was used for generating rarefaction curves and R version 1.3 was used for alpha-diversity indices analyses. ***t***-test was used to ascertain differences in the indices.

### Identification of Differentially Abundant Taxonomic Groups

Kruskal*–*Wallis and Wilcoxon signed rank tests (Benjamini Hochberg [BH] corrected *p* < 0.05) were used for identifying the differentially abundant taxonomic groups both at the phyla and genera level in the mycobiomes. Consequently, the rank-normalized abundances of the discriminatory genera (scaled between 0 and 1) were clustered in two-dimensional heatmaps using R. Non-metric multidimensional scaling (NMDS) plots using Canberra dissimilarity were generated to visualize the abundance differences at the OTU level between the mycobiomes (51).

### Interaction Networks Between Fungal Genera in the Mycobiomes

Interaction networks between the genera in HC-SW and BK-SW mycobiomes were generated using CoNet (52) in Cytoscape (42) using Spearman pairwise correlation coefficient (***r***).

## Results

The 26 HC and 22 BK individuals recruited in this study were age and gender matched (*p* ≥ 0.05). The mycobiome data of a subset of HC (*n* = 21 out of 26) were published earlier (14, 15).

### Identification of Fungi Through Culture Method

Fungi were detected only in a small number of conjunctival swabs of HC (3 of 52 swabs; 5.77%). Aspergillus was the only genus identified and the species included Aspergillus flavus (HC01.RE and HC28.RE) and Aspergillus niger (HC02.LE). Fungi were not detected in any of the corneal scrapings of the BK patients (see **Supplementary Table S1**).

### NGS Analysis of the Ocular Mycobiomes

**Figure 1:**
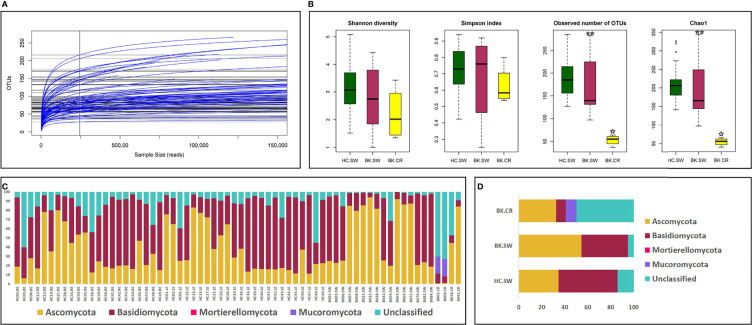
**(A)** Rarefaction analysis and **(B)** alpha-diversity indices of mycobiomes from the conjunctivae and corneal scrapings in the three cohorts of HC-SW, BK-SW, and BK-CR. The observed number of OTUs and Chao1 index was statistically significant (p < 0.05, BH corrected) between HC-SW and BK-CR (indicated by a single asterisk) and between BK-SW and BK-CR (indicated by two asterisks) by Wilcoxon test. Abundance **(C)** and mean abundance **(D)** of different fungal phyla in the three cohorts.

Mycobiomes were generated in majority of the ocular surface samples (67 of 96 samples), which included 46 from HC-SW, 16 from BK-SW, and 5 from BK-CR. The mycobiome of BK03.CR was excluded from the analysis since the reads were less than 5,000. Corneal scrapings from 17 other BK patients after the use for the detection of cultivable fungi were not sufficient in quantity for DNA extraction and the subsequent NGS analysis. The 66 mycobiomes generated a total of 16.27, 9.17, and 1.6 million average HQ reads respectively, and the average number of reads per mycobiome was highest in BK-SW (0.57 million) followed by BK-CR (0.4 million) and HC-SW (0.35 million). The average percentage of the HQ reads that could be assigned to an OTU was 95.57%. In the three cohorts, a total of 897 OTUs (20 reference-OTUs and 877 denovo-OTUs) were identified (see **Supplementary Table S2**). The 66 mycobiomes exhibited saturation of the reads in the rarefaction curves indicating reasonable sequencing depth and coverage in the mycobiomes (**Figure 1A**).

### Alpha-Diversity Indices of the Ocular Fungal Microbiomes

Alpha-diversity analysis indicated that the number of observed OTUs and Chao1 index (richness) was statistically significantly based on Kruskal–Wallis test when the diversity was compared between HC-SW, BK-SW, and BK-CR. Wilcoxon test also confirmed that the number of observed OTUs and Chao1 index was statistically significant between HC-SW and BK-CR and between BK-SW and BK-CR mycobiomes (**Figure 1B**).

### Fungal Phyla Inhabiting the Ocular Surface of HC and BK Patients

Fungi affiliated to the phyla Ascomycota and Basidiomycota were present in all the analyzed mycobiomes. Basidiomycota was the most dominant phylum followed by Ascomycota in HC-SW, whereas in BK-SW and BK-CR, Ascomycota was the predominant phylum followed by Basidiomycota. Mucoromycota was a minor phylum and was not consistently observed in all the HC-SW, BK-SW, and BK-CR mycobiomes and were significantly different between the cohorts. Another minor phylum, Mortierellomycota, was detected only in a few HC-SW and BK-SW mycobiomes and was also significantly different between them. The mean abundance of unclassified reads was more in BK-CR (49.92%) compared to HC-SW (14.08%) and BK-SW (5.09%) mycobiomes (**Table 1** and **Figures 1C** **, D**).

**Table 1:** Differences in the abundance (%) of fungal phyla in the mycobiomes of the conjunctivae of healthy controls (HC-SW, *n* = 46) and in the conjunctivae (BK-SW, *n* = 16) and corneal scrapings of bacterial keratitis patients (BK-CR, *n* = 4).

S. No.	Phylum	HC-SW			BK-SW			BK-CR			Wilcoxon test *p*-value (BH corrected)			
		Abundance		Present out of 46 eyes sampled	Abundance		Present out of 16 eyes sampled	Abundance		Present out of 4 eyes sampled	HC.SW vs.BK.SW vs.BK.CR	HC.SWvs.BK.SW	HC.SWvs.BK.CR	BK.SW vs.BK.CR
		Mean	Range		Mean	Range		Mean	Range					
1	Basidiomycota	51.27	14.13–85.22	46	40.21	4.8–78.94	16	8.2	6.84–10.63	4	0.002	0.103	0	0.04
2	Ascomycota	34.61	6.29–83.11	46	54.61	18.86–94.1	16	32.59	0.56–84.11	4	0.002	0.002	0.825	0.298
3	Mucoromycota	0.04	0–0.86	2	0.01	0–0.13	6	9.3	0–18.61	2	0.002	0.002	0.002	0.311
4	Mortierellomycota	0	0–0	1	0.08	0–0.39	5	0	0–0	0	0.002	0.002	0.825	0.298
5	Unclassified	14.08	2.72–60.33	46	5.09	0.11–31.33	16	49.92	9.05–73.13	4	0	0	0.017	0.01

### Abundance Differences in the Fungal Genera in the Conjunctivae and Corneal Scraping Mycobiomes of BK Patients

**Figure 2:**
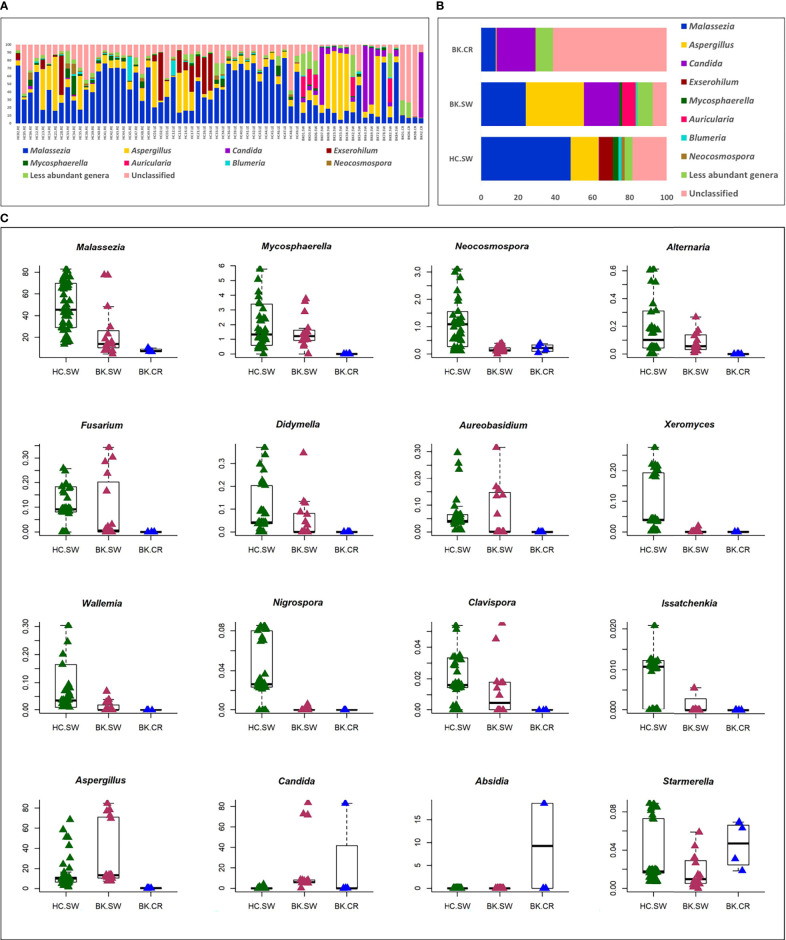
Differences in the abundance **(A)** and mean abundance **(B)** of fungal genera in the mycobiomes of the conjunctivae of healthy controls (HC_SW), conjunctivae of keratitis individuals (BK-SW), and corneal scrapings of keratitis individuals (BK_CR). Genera with mean abundance <1% were designated “less abundant genera”. In **(C)**, candidate fungal genera exhibiting significant differential abundance (Wilcoxon test, BH corrected p < 0.05) with an abundance of >1% in at least any one of the above cohorts is depicted. Median abundances (horizontal line) and inter-quartile ranges are indicated in the plots.

A total of 116 genera were identified in the 66 ocular mycobiomes (see **Supplementary Table S3**). Malassezia, Aspergillus, Neocosmospora, Candida, Starmerella, Cladosporium, and Saccharomyces were shared between the 3 cohorts (**Figures 2A, B**). The conjunctival mycobiomes of HC (HC-SW) shared 78 genera with the conjunctivae (BK_SW) and 7 genera with the corneal scrapings of the keratitis patients (BK-CR), whereas between BK-SW and BK-CR, only the genus Absidia was shared (See **Supplementary Table S3**). Despite these similarities, the three cohorts could be differentiated based on the abundance of the genera, which either significantly decreased (10 and 13 genera, respectively) or increased (2 and 1, respectively) in abundance in BK-SW and BK-CR mycobiomes compared to HC-SW (**Tables 2**, **3** and **Figure 2C**). Between BK-CR and BK-SW, only four genera significantly decreased in abundance (**Table 4** and **Figure 2C**).

**Table 2:** Significant median abundance differences (BH corrected *p* ≤ 0.05) in the fungal genera in the mycobiomes of the conjunctivae of healthy controls (HC-SW, *n* = 46) and bacterial keratitis patients (BK-SW, *n* = 16).

S. No.	Genus	Median abundance (%)		Wilcoxon test *p*-value (BH-corrected *p*-value ≤ 0.05)†	Pathogenicity
		HC-SW	BK-SW		
**Genera decreased in BK-SW**					
1	*Malassezia*	45.421	13.795	0	Animal/Human pathogen (53)
2	*Neocosmospora*	1.09	0.136	0	Plant/Animal/Human pathogen (54)
3	*Xeromyces*	0.039	0	0	Not known
4	*Wallemia*	0.036	0	0	Human pathogen (55)
5	*Nigrospora*	0.026	0	0	Plant/Human pathogen (56)
6	*Issatchenkia*	0.011	0	0	Plant/Human pathogen (57)
7	*Microascus*	0.005	0	0	Plant/Human pathogen (58)
8	*Strelitziana*	0.003	0	0	Not known
9	*Trichosporon*	0.002	0	0	Human pathogen (59)
10	*Exserohilum*	0.001	0	0	Plant/Human pathogen (60)
**Genera increased in BK-SW**					
1	*Aspergillus*	9.852	13.337	0	Human pathogen (61)
2	*Candida*	0.071	6.188	0	Human pathogen (62)

^†^p-value of 0 indicates ≤ 0.0001.

**Table 3:** Significant median abundance differences (BH-corrected *p* ≤ 0.05) in the fungal genera in the mycobiomes of the conjunctivae of healthy controls (HC-SW, *n* = 46) and corneal scrapings of bacterial keratitis patients (BK-CR, *n* = 4).

S. No.	Genus	Median abundance (%)		Wilcoxon test *p*-value (BH-corrected *p*-value ≤ 0.05)†	Pathogenicity
		HC-SW	BK-CR		
**Genera decreased in BK-CR**					
1	*Malassezia*	45.421	7.424	0	Animal/Human pathogen (53)
2	*Aspergillus*	9.852	0.42	0	Human pathogen (61)
3	*Mycosphaerella*	1.323	0	0	Plant pathogen (63)
4	*Alternaria*	0.102	0	0	Plant/Human pathogen (63)
5	*Fusarium*	0.091	0	0	Plant/Animal/Human pathogen (64)
6	*Didymella*	0.042	0	0	Plant pathogen (65)
7	*Aureobasidium*	0.041	0	0	Human pathogen (66)
8	*Xeromyces*	0.039	0	0	Not known
9	*Wallemia*	0.036	0	0	Human pathogen (55)
10	*Nigrospora*	0.026	0	0	Plant/Human pathogen (56)
11	*Clavispora*	0.016	0	0	Human pathogen (67)
12	*Issatchenkia*	0.011	0	0	Plant/Human pathogen (57)
13	*Strelitziana*	0.003	0	0	Not known
**Genus increased in BK-CR**					
1	*Absidia*	0	9.287	0	Animal/Human pathogen (68)

^†^p-value of 0 indicates ≤ 0.0001.

**Table 4:** Significant median abundance differences (BH-corrected *p* ≤ 0.05) in the fungal genera in the mycobiomes of the conjunctivae (BK-SW, *n* = 16) and corneal scrapings of bacterial keratitis patients (BK-CR, *n* = 4).

S. No.	Genus	Median abundance (%)		Wilcoxon test p-value(BH-corrected *p*-value ≤ 0.05)†	Pathogenicity
		BK-SW	BK-CR		
**Genera decreased in BK-CR**					
1	*Aspergillus*	13.337	0.42	0	Human pathogen (61)
2	*Mycosphaerella*	1.212	0	0	Plant pathogen (63)
3	*Blumeria*	0.633	0	0	Plant pathogen (69)
4	*Alternaria*	0.056	0	0	Plant/Human pathogen (63)

**^†^** p-value of 0 indicates ≤ 0.0001.

**Figure 3:**
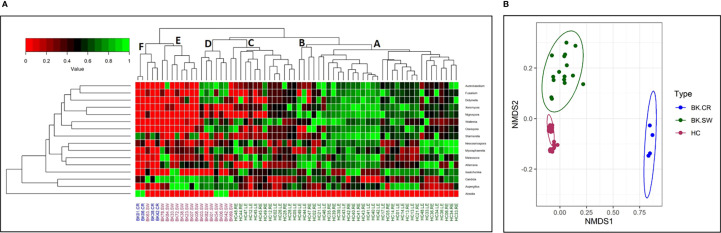
**(A)** Two-dimensional heatmap analysis of 16 differentially abundant fungal genera as determined by Kruskal–Wallis test in the mycobiomes of conjunctivae of healthy controls (HC-SW; green), conjunctivae of bacterial keratitis individuals (BK-SW; red), and corneal scrapings of bacterial keratitis individuals (BK-CR; blue). The genera were rank normalized (scaled between 0 and 1) based on abundance, and the median abundance of the genera was >0.01% in at least one group of samples. Discriminating genera were arranged based on hierarchical clustering. In **(B)**, beta-diversity analysis using NMDS plots based on Canberra dissimilarity of fungal OTU abundance was used to demonstrate that the fungal communities varied significantly across HC-SW, BK-SW, and BK-CR (PERMANOVA, p = 0.001).

The 16 differentially abundant fungal genera in the 3 cohorts were also analyzed by two-dimensional heatmap (**Figure 3A**) using the rank-normalized abundances (scaled between 0 and 1). The analyses sufficiently segregated the HC-SW, BK-SW, and BK-CR mycobiomes. The 46 mycobiomes of the conjunctivae of HC (HC-SW) formed 3 sub-clades (A, B, and C), whereas majority of BK-SW mycobiomes (14 of 16) clustered into sub-clades D and E and the remaining 2 mycobiomes clustered with HC-SW and BK-CR mycobiomes in sub-clades C and F. The 4 BK-CR mycobiomes clustered into one sub-clade F. The segregation of the ocular mycobiomes of the 3 cohorts was also confirmed using NMDS plots based on Canberra dissimilarity of fungal OTU abundances (p = 0.001) (**Figure 3B**).

A few of the BK patients had taken either topical or oral antibiotics and therefore were analyzed after categorizing the BK samples into treated (BK-SW-T, n = 5; BK-CR-T, n = 1) and untreated (BK-SW-UT, n = 11; BK-CR-UT, n = 3) groups. Wilcoxon test indicated that the mycobiomes of BK-SW and BK-CR were similar irrespective of whether they had taken antibiotics or not.

### Interactions Between the Fungal Genera Inhabiting the Ocular Surface of HC and BK Patients

**Figure 4:**
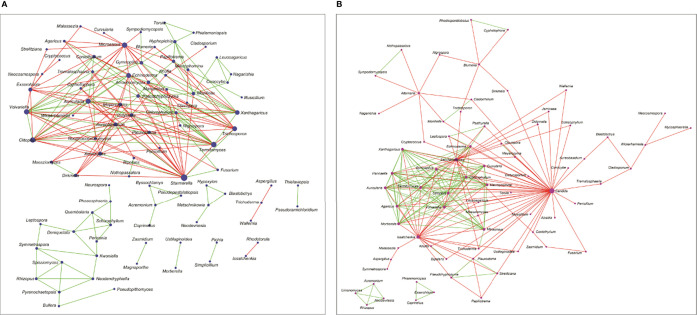
Interaction networks between the genera in the mycobiomes of conjunctivae of healthy controls (HC-SW, n = 46) **(A)** and bacterial keratitis patients (BK-SW, n = 16) **(B)**. (The size of the nodes in the network correspond to their degree of interaction. Green edges correspond to positive correlations/interactions and red edges correspond to negative correlations/interactions.).

Based on pairwise correlations of abundance of fungal genera (**Figures 4A, B**), it was observed that the mycobiomes of the conjunctivae in both the HC (HC-SW) and BK individuals (BK-SW) are different (**Figures 4A, B**). The HC-SW network had both positive and negative interactions, whereas the BK-SW network had more negative interactions. Several *“*hub*”* genera (with *>*10 interactions) were observed in conjunctivae of HC (12) and BK patients (17). In HC-SW mycobiomes, six *“*hub*”* genera, namely, Starmerella, Microascus, Trichosporon, Xeromyces, Exserohilum, and Aureobasidium, were unique to HC-SW, whereas the remaining 6 *“*hub*”* genera (Termitomyces, Clitopilus, Volvariella, Auricularia, Echinoderma, and Xanthagaricus) were shared with BK-SW. The genera Candida, Saccharomyces, Issatchenkia, Gymnopilus, Marasmius, Mortierella, Chlorophyllum, Hannaella, Curvularia, Macrophomina, and Agaricus were found to be the unique hubs in BK-SW mycobiomes. We did not generate an interaction network for BK-CR mycobiomes since the number of genera were extremely less.

## Discussion

In this study, two dominant phyla, Ascomycota and Basidiomycota, and one other phylum, Mucoromycota, were detected in the mycobiomes of the conjunctivae of both healthy individuals and individuals with BK and in the corneal scraping of individuals with BK, thus confirming earlier studies (14, 15). Furthermore, 111 different genera were identified in conjunctival mycobiomes of healthy control, out of which 10 genera, namely, *Malassezia*, *Aspergillus, Mycosphaerella, Neocosmospora, Aureobasidium, Walemmia, Xeromyces, Starmerella, Issatchenkia*, and *Streilitziana*, were present in all the eyes of HC-SW and constituted the core ocular mycobiome according to the criteria of Turnbaugh et al. (70). Earlier studies had indicated that *Aspergillus, Setosphaeria, Malasezzia*, and *Haematonectria* (15) constituted the core mycobiome in the conjunctival swabs of healthy individuals. The discrepancy between the studies may be attributed to variations between individuals with respect to age, the region of their origin, and the pathology (71–73). Furthermore, in accordance with earlier studies (5, 15, 74, 75), fungi were detected in the conjunctival swabs of only 5.77% of HC individuals and *Aspergillus* was the only genus identified. Fungi were not detected in the corneal scrapings of the BK patients though corneal scrapings from fungal keratitis patients were positive for fungi (14). The results also indicated that in the NGS method, many more fungi were detected compared to the conventional cultivable method.

Dysbiosis in the gut microbiome has been implicated in intestinal and extraintestinal diseases (1) such as immune-mediated diseases (76), inflammatory diseases (77, 78), cancers and mental disorders (79), and also ocular diseases like uveitis (18, 19, 80), age-related macular degeneration (81), bacterial and fungal keratitis (20, 82), and diabetic retinopathy (21, 22). However, compared to gut microbiome studies, studies implicating the dysbiosis in ocular microbiome/mycobiome in ocular disease are few, which include blepharitis (23), contact lens wearers (24), Stevens–Johnson syndrome (25, 83), BK (27), and fungal keratitis (14, 26).

Results from the present study indicated that the ocular mycobiomes of the ocular surface including mycobiomes from the conjunctivae and corneal scrapings from the conjunctivae of HC (HC-SW), conjunctivae of BK individuals (BK-SW), and corneal scrapings of BK individuals (BK-CR) could be differentiated based on the number of observed OTUs and Chao1 index indicating richness of the mycobiomes (**Figure 1B**). In addition, the cohorts differed with respect to the abundance of two dominant fungal phyla, Basidiomycota and Ascomycota (**Table 1**), and several genera that were either increased or decreased in abundance in the keratitis ocular surface compared to conjunctival mycobiomes of the unaffected individuals (HC-SW) (**Tables 2** and **3**). Heatmap and beta-diversity analysis segregated the mycobiomes of the conjunctivae of HC from the mycobiomes of the conjunctivae and corneal scrapings of BK patients (**Figures 3A, B**). The above distinct significant changes in the fungal communities across HC-SW, BK-SW, and BK-CR imply dysbiosis (alterations in the diversity and abundance) in the ocular surface mycobiome (conjunctivae and corneal scrapings) of BK individuals compared to control individuals. In an earlier study, dysbiosis in the mycobiome was reported in fungal keratitis individuals (14). In this study, the Institutional Ethics Committee did not approve collection of corneal scrapings from HC since such a collection of corneal scraping of HC would damage the normal cornea of a healthy individual.

The implication of mycobiome dysbiosis in BK could be better understood based on the discriminating genera in keratitis patients compared to the HC (**Tables 2** and **3**). Two abundant genera, namely, *Malassezia* (45.421% to 13.795%) and *Neocosmospora* (1.09% to 0.1346%), along with 9 other minor genera (<1.0%) decreased in abundance in BK-SW, whereas the major genera *Malassezia*, *Aspergillus*, and *Mycosphaerella* and 10 other minor genera (<1% abundance) decreased in BK-CR compared to HC-SW. All these genera are reported to be plant/animal/human pathogens and may be inflammatory, and thus, their decrease is difficult to interpret. However, the simultaneous increase in abundance of *Aspergillus* and *Candida* in BK-SW and *Absidia* in BK-CR, which are human pathogens, may support the inflammatory status of keratitis. In fact, *Aspergillus* keratitis is an important ophthalmological problem across the world (84–86). *Candida* is also a common causative agent of keratitis, and different species of *Candida* such as *Candida albicans, C. krusei*, *C. fermentati*, *C. famata*, *C. glabrata*, C*. tropicalis*, *C. parapsilosis*, and *C. guilliermondii* have been reported to be associated with the eye of individuals with keratitis (61, 87–89). Species of *Absidia*, like *Absidia corymbifera*, caused keratitis in an immunocompetent male patient with no corneal injuries (90) and also in individuals following trauma (91, 92). It was also reported that *A. corymbifera* caused fungal keratitis that led to endophthalmitis (93). When the median abundance of the genera was compared between BK-SW and BK-CR, only four genera were decreased in abundance, implying that they were probably not important to support the keratitis state. This in fact may not hold good for *Aspergillus*, which is known to cause keratitis as discussed above. The remaining 3 genera, *Mycosphaerella, Alternaria*, and *Blumeria*, which decreased in abundance, are minor phyla and may not be implicated in keratitis. In fact, *Alternaria* spp. is an uncommon cause of mycotic keratitis (94) whereas *Mycosphaerella* and *Blumeria* are plant pathogens and little is known about their ability to cause keratitis (95, 96).

Interaction networks also indicated that the mycobiomes of HC-SW and BK-SW are different (**Figures 4A, B**) with the BK-SW network, exhibiting more negative interactions than the HC-SW network. The two networks shared six genera (*Termitomyces, Clitopilus, Volvariella, Auricularia*, *Echinoderma*, and *Xanthagaricus*), and these may not specifically influence the keratitis state. However, *Candida, Saccharomyces, Issatchenkia, Gymnopilus, Marasmius, Mortierella, Chlorophyllum, Hannaella, Curvularia*, *Macrophomina*, and *Agaricus* were found to be the unique hubs in BK-SW mycobiomes and may positively influence keratitis. Five of these, *Candida* (61, 87–89), *Saccharomyces* (97), *Mortierella* (98), *Curvularia* (99), and *Macrophomina* (100), have been reported to be causative agents of keratitis. The remaining unique genera **(** *Agaricus, Gymnopilus, Marasmius, Chlorophyllum*, and *Hannaella* **)** exhibited positive or negative interactions. In these networks, it was observed that in keratitis individuals, certain genera exhibit positive interactions with other pathogens, and such genera are likely to support the inflammatory status in the keratitis state.

Earlier studies had indicated dysbiosis in the ocular (conjunctival, corneal, fluid, tears, eyelash, etc.) bacterial microbiomes in the diseased eye as in conjunctival scarring (101), dry eye disease (102), contact lens-associated inflammation (24), Stevens**–**Johnson syndrome (25), blepharitis (23), Keratitis (26), and endophthalmitis (103). One study also reported dysbiosis in the fungal microbiome in individuals with fungal keratitis (14).

Dysbiotic changes in the gut microbiomes and mycobiomes have been implicated as causative changes in patients with ocular diseases like uveitis (18, 19), bacterial and fungal keratitis (20), and diabetic retinopathy (21, 22). A few studies have also reported the involvement of the ocular surface microbiome in the pathogenesis of ocular diseases (23–29), which would be relevant to the current study. For instance, Lee et al. (23) identified high abundance of commensal bacteria in ocular samples of both HC and blepharitis patients, but their compositions were different depending on the occurrence of blepharitis. It was suggested that the abundance of the commensal bacteria would ultimately influence blepharitis, which is dependent on the interaction of the ocular microbial community with the eye. On similar lines, Shin et al. (24) suggested that, in contact lens wearers, the commensals of the ocular surface are also important because in the absence of lenses, commensals interact with the host immune system and suppress microbial pathogenicity, thus overcoming conjunctivitis and keratitis. In SJS patients, the ocular surface is occupied by more diverse microorganisms with increased proportion of opportunistic pathogens (25), thus influencing chronic inflammation and opportunistic infections. In contrast, the conjunctival microbiome in fungal keratitis individuals exhibited decreased bacterial diversity along with increase in pathogenic bacteria, which probably influence the pathogenesis of FK (26). A consistent increase in the abundance of pathogenic bacteria was also observed in the conjunctiva of BK patients (27). Our group has also published two papers on ocular surface mycobiomes related to fungal keratitis patients (14) and post-fever retinitis patients (104). In the former, alterations in the fungal microbiota were observed with respect to both diversity and abundance, and the conjunctival fungal community varied significantly in the HC compared to the cornea of the keratitis patients. Furthermore, it was predicted that the unique genera in the fungal keratitis patients in both the conjunctivae and corneas were opportunistic pathogens or pathogens (14). Mycobiome analysis in the vitreous of post-fever retinitis individuals also showed a significant increase in the genera that are pathogenic (104). Thus, compared to microbiome studies on ocular surface (conjunctivae, corneas, or both), only limited data are available on mycobiome changes in the diseased eye. However, increase in abundance and increase in pathogens in the diseased state may be related to causing or exacerbating ocular surface inflammation as in keratitis.

This is the first report on dysbiosis in the ocular surface mycobiomes of BK patients compared to HC devoid of any ocular disease.

## Conclusions

- The study reports dysbiosis in the mycobiome of the ocular surface (conjunctivae and corneal scrapings) in BK patients compared to the conjunctivae of HC.
- Mycobiomes were altered in diversity and abundance at both the phylum and genera level.
- Both alpha- and beta-diversity analysis confirmed dysbiosis in the ocular mycobiome in BK patients.
- Ten genera, namely, *Malassezia*, *Aspergillus, Mycosphaerella, Neocosmospora, Aureobasidium, Walemmia, Xeromyces, Starmerella, Issatchenkia*, and *Streilitziana*, constituted the core ocular mycobiome.
- A metagenome approach may help to unravel the functions of the discriminating genera on the ocular surface of keratitis individuals.
- Longitudinal studies would unravel the dynamics of the microbiota with progression of the disease.

## Limitations of the Study

- The mycobiome of corneal scrapings need to be analyzed further to realize whether the subjects would exhibit any further diversity.
- The mycobiome analysis is insufficient to identify the diverse fungi to the species level and this is important for functional interpretation of the data with reference to the pathogenesis of the fungi.
- Though our results highlight dysbiosis in the ocular surface mycobiome in keratitis individuals compared to the controls, further animal experiments using inflammatory models of animals could shed further light on the susceptibility of cornea and conjunctivae to alterations in the mycobiomes.
- More studies on mycobiomes would help also to interpret the data with respect to the involvement of commensals in pathogenesis.
- Compliance of patients to the collection protocol is a common limitation.
- Longitudinal studies would unravel the dynamics of the mycobiome with progression of the disease.

## Data Availability Statement

Data relevant to this study are available in the NCBI database, Bio project accession number: PRJNA818470.

## Ethics Statement

The studies involving human participants were reviewed and approved by the Institutional Review Board of L V Prasad Eye Institute. The study was conducted in accordance with the Research Review Board and Ethics Committee of LVPEI, Hyderabad (Ethics Ref. No. LEC 06-14-060). The patients/participants provided their written informed consent to participate in this study.

## Author Contributions

Conceptualization: SiS. Data curation: RJ, SC, and GP. Formal analysis: RJ and GP. Funding acquisition: SiS. Investigation: RJ and SC. Methodology: SiS. Project administration: SiS. Resources: SaS, PG, SM, and SiS. Software: RJ and GP. Supervision: SiS. Validation, RJ. Visualization: RJ and GP. Writing—original draft: RJ and SiS. Writing—review and editing: RJ and SiS. All authors contributed to the article and approved the submitted version.

## Funding

This work was supported by the Department of Biotechnology, Ministry of Science and Technology (grant number DBT: BT/PR32404/MED/30/2136/2019). The sponsor or funding organization had no role in the design or conduct of this research.

## Conflict of Interest

The authors declare that the research was conducted in the absence of any commercial or financial relationships that could be construed as a potential conflict of interest.

## Publisher’s Note

All claims expressed in this article are solely those of the authors and do not necessarily represent those of their affiliated organizations, or those of the publisher, the editors and the reviewers. Any product that may be evaluated in this article, or claim that may be made by its manufacturer, is not guaranteed or endorsed by the publisher.

## Supplementary Material

Supplementary Table 1 Details of healthy controls (*n* = 26) and bacterial keratitis patients (*n* = 22).

Supplementary Table 2 Relative abundances of fungal OTUs (>0.001% of high-quality reads) from 66 ocular mycobiomes from conjunctivae of healthy controls (HC-SW, *n* = 46) and conjunctivae (BK-SW, *n* = 16) and corneal scrapings of bacterial keratitis patients (BK-CR, *n* = 4).

Supplementary Table 3 Abundance (%) of fungal genera (>0.001% of high-quality reads) in the 66 ocular mycobiomes from conjunctivae of healthy controls (HC-SW, *n* = 46; RE = 22, LE = 24) and conjunctivae (BK-SW, *n* = 16) and corneal scrapings of bacterial keratitis patients (BK-CR, *n* = 4).
